# Behind every great research project is great data management

**DOI:** 10.1186/s13104-022-05908-5

**Published:** 2022-01-21

**Authors:** Samantha Kanza, Nicola J. Knight

**Affiliations:** Department of Chemistry, Faculty of Engineering and Physical Sciences, University of Southampton, University Road, Southampton, SO17 1BJ UK

**Keywords:** Research data management, Data management plans, Data organisation, Data sharing, FAIR data, Data ethics, Reproducibility

## Abstract

Research data management (RDM) is the cornerstone of a successful research project, and yet it often remains an underappreciated art that gets overlooked in the hustle and bustle of everyday project management even when required by funding bodies. If researchers are to strive for reproducible science that adheres to the principles of FAIR, then they need to manage the data associated with their research projects effectively. It is imperative to plan your RDM strategies early on, and setup your project organisation before embarking on the work. There are several different factors to consider: data management plans, data organisation and storage, publishing and sharing your data, ensuring reproducibility and adhering to data standards. Additionally it is important to reflect upon the ethical implications that might need to be planned for, and adverse issues that may need a mitigation strategy. This short article discusses these different areas, noting some best practices and detailing how to incorporate these strategies into your work. Finally, the article ends with a set of top ten tips for effective research data management.

## Introduction

A research project without proper research data management (RDM) is akin to building a house without laying the foundations. Good RDM is critical to the success of a research project; however, it is an element that is often neglected even when required by funding bodies. Planning your RDM needs to take into account not just the current moment, but considering how you will look back on your information in several years or how you might be able to share the information with colleagues, possibly across multiple disciplines, in a form that they can easily understand. There are many aspects involved in the data research lifecycle that will help a research project and its data to be findable, accessible, interoperable and re-useable (FAIR) [1]. In this short article we are going to discuss some of the key areas involved in RDM including: Organising, storing and sharing your data, creating data management plans and ensuring that any research conducted is both ethical and reproducible. We discuss why these areas are important and how they might be incorporated in your work and conclude with a list of our top ten tips for how to manage your research data.

## Main text

## Data organisation and storage

How you organise and store your data will shape your capacity to find, manage, publish and re-use it at a later date [2]. The first person likely to benefit from a sensible organisational system is your future self. Using sensible easy to follow folder and file structures will enable you to easily locate different pieces of your data. Just randomly naming files and putting in a haphazard folder structure will not benefit either you or anyone looking to use your data in the future. Another potential consideration is being aware of restrictions on where you are permitted to store your research data, such as institutional requirements or specific collaborator requirements related to security and international transfer of data.

This should be something that you think about at the very beginning of your work, as it is much simpler to add to an existing structure than having to go back and rework years of files at the end of the project (if you can even remember what each file referred to). If you are storing lots of similar types of data then you might want to consider making template folders that you can use each time you create a new dataset. When working in a collaborative research project it is also important that the organisational strategies are agreed upon as a group at the start to ensure consistency across the team with respect to both where and how team members are storing and organising the project data.

It is also worth identifying which aspects of your data you will need to store for the short term and long term, and how you are going to store the data. Further, it is worth considering the trade-off between data storage and recreating data, as data that is expensive to store but easy to re-create doesn’t necessarily need to be stored [3].

If your data is being produced in a proprietary format, then you need to work out how to store it in an accessible way that you and others can use it later down the line even if you don’t have access to the software that produced it. One way of ensuring the longevity of your data is to save it in a .txt file, meaning that even if the proprietary files become unusable, the data still remains in a readable and editable format. However, this strategy should only be employed alongside saving the data in the original data formats as opposed to relying on this as the primary storage method. Additionally, when doing this, it is important to supply context alongside the data potentially in the form of a README data description file or as additional metadata as merely saving the data in raw text files isn’t very helpful when it comes to sharing or understanding it later down the line.

It is also worth remembering that if you are working on collaborative research projects, then a key aspect of organising your data is communication. Even with the most organised group members, projects and data cannot be effectively managed unless all group members are communicating and have agreed on who is doing what with the different pieces of data [4]. The more data, and the more complex the data, the more time you need to devote to planning the organisation and communication of the data.

## Data management

Poor data management can lead to data breaches and subsequently, unsuccessful and potentially harmful research projects. Managing your data well and planning how to achieve this from the start is absolutely key to a successful research project and is often a requirement of Research Application Funding Bodies. In order to achieve this, a Data Management Plan (DMP) should be created which outlines how the data is going to be managed throughout the entire project lifecycle. Many universities have their own internal resources for creating these plans, but there are also templates available from DMP Online [5].

A DMP should be an active document that is referred to throughout the project and used to measure whether it is on track. It is essential that these documents are updated throughout the project to reflect any changes, and data managers should be consulted with respect to the DMP throughout the entire project lifecycle, rather than only at the beginning. Further, in collaborative data projects these plans become even more essential as all group members should be collecting and handling data in the same way.

Another core aspect of data management is version control. All the work associated with a research project should be backed up, but versions should also be kept such that changes can be recorded and documents or data can be rolled back to a previous version if necessary. There are a number of ways to version data depending on the nature of the data and the project teams expertise. There are version control systems such as GitHub [6] for code or datasets. For documents, you can use integrated track changes, alternatively separate files can be created with version numbers, or version control tables can be added at the top of documents to record in document changes.

## Data publication and sharing

Publishing your data and making it shareable is an important outcome for any successful research project. Ultimately it is desirable to disseminate the useful and relevant outputs of a research project to those who might be interested in reading or using them. It is important to give careful consideration to what parts of the project are published, and where they are published. Ultimately, the choice of what to publish and where to publish might be a requirement of the research funding, in which case that should be adhered to. If there is no specific data mandate, then publishing data in an open access repository that is relevant and well used within the research domain is advisable.

Data should always be published with suitable metadata, README files, and it should adhere to the FAIR standards of being findable, accessible, interoperable and re-useable [1]. Ideally, the datasets would have their own DOI and would be published under a license that enables their re-use. When considering what extent of the data and descriptions to publish, it is worth establishing what level of data (and methods) needs to be shared in order for the project results to be reproduced. Depending on the nature of your data (e.g. if it contains any sensitive or personal data) it may be necessary to anonymise and or aggregate the data before making it available [7].

## Data reproducibility

Reproducibility (or the lack thereof) is a significant problem in scientific research in the 21st Century, as in order to allow other scientists to assess your work and also use it in the advancement of scientific knowledge it is crucial that the work can be reproduced. There are unfortunately a large number of peer reviewed scientific studies that are not reproducible [8, 9], which could be due to lack of availability of raw data, poor methodological explanations, missing data, and a number of other considerations. There are so many factors, parameters and methods that can be used on data, right from the point of acquisition, through analysis, up to the visualisation of outputs. All of these changes can affect the data findings, and it essential that these are all captured alongside the data if it is to stand a chance of being reproducible.

There are a number of steps that can be taken to aid with facilitating reproducibility. One of the crucial elements in reproducibility is sharing your data provenance, that could be pointing researchers to specific datasets, if using existing data, or your detailed protocols if you collected the data yourself. It is also becoming increasingly important to share your code and detailed methods alongside the data used in the analysis, as this enables other researchers to understand the processes that were undertaken and attempt to reproduce them. It is worth considering using an electronic lab notebook (ELN) System or a notebook that can combine commentary and analysis code such as Jupyter Notebooks [10]. Additionally, using version control systems for your documents and code will allow others to see any changes that you have made, and specific versions can be viewed and used. Ultimately, if you yourself could not reproduce the project results from the data and documents that you have shared, then you cannot expect another researcher to be able to. This is something that should be considered and evaluated before finalising what data to share.

## Data ethics

Data ethics is another vital aspect of responsible research. In most jurisdictions any study that involves humans (whether through direct data generation, the use of their tissue/cells, or the use of their previously generated data e.g. tweets or online contributions) will need specific ethics approval [11], but ethical considerations should apply generally as well.

The core requirements of an ethics application are to lay out the purposes of the study, what data you are collecting and why, and to ensure that participants are fully informed about their involvement [12]. Consent needs to be obtained from any active participants (and that includes the researchers themselves), and researchers need to make a data protection plan and devise a risk assessment to ensure that the research is being conducted safely, with mitigated risks, and that the data collected is going to be adequately protected.

If you are working with personal data then there are ethical considerations around collection of the appropriate amount of data to collect and ensuring that it is anonymised as soon as possible. It is also worth remembering that, even when projects are not working with personal data there are ethical considerations around the potential effects of your research and possible unintended consequences to the communities involved [13].

## Outlook

The biggest piece of advice that we can give to improve your data management is start early! Don’t leave thinking about this until the project wraps up, or when writing up your results, think about it when you start out, and continue to evolve this as your project matures. Don’t be afraid to ask for advice, as there is lots of expertise out there, and remember that changes can always be made. It is obviously preferable to start out with an optimum data management plan, but it is much better to change a system that isn’t working or to make improvements than to just stick to a plan that isn’t working. Making small steps towards better overall data handling is better than not taking any steps.

**Fig. 1:**
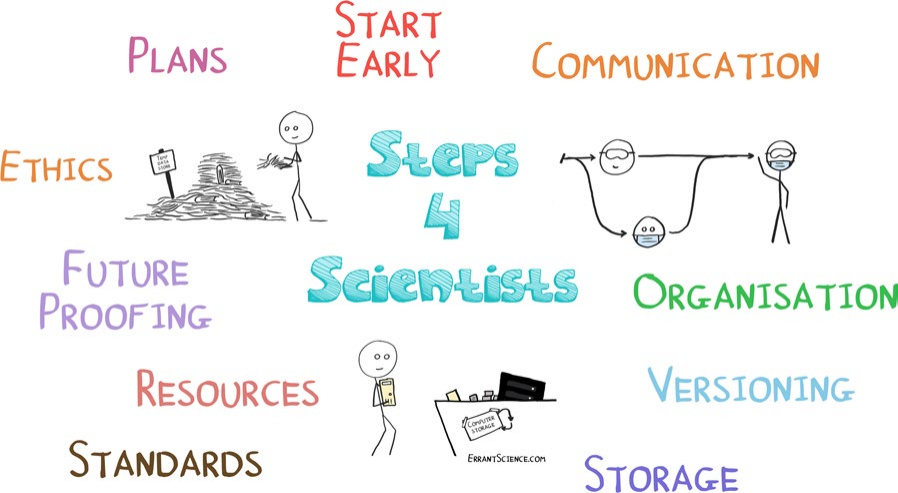
Top ten tips for good research data management

To help with this here are our top ten tips for good research data management as shown in Fig. 1.

1. Start early: Plan your data management strategy right from the start, think about every aspect of your project from the data collection, organisation, storage, and even where you are planning on publishing and sharing the results.
2. Data management plan: Make one of these right at the beginning and refer to it and improve it throughout the entire project life cycle.
3. Organisation is key: Use sensible folder/file structures that have been agreed with the entire team.
4. Version control your work: Decide on what version control systems you are going to use and implement these plans from the beginning.
5. Storage strategy: Consider your long term and short term data storage. And implement the 321 data storage rule: (3 copies of the data, within 2 types of media, with 1 stored at a separate site), and NEVER rely on USB sticks.
6. Remember your standards and be FAIR: Think about what standards you are going to make your data available in. Data should be Findable, Accessible, Interoperable and Re-useable.
7. Consider ethics: If you are interacting with human data in any way, you will need ethics! These applications can take a while to write and obtain approval for, so start straight away!
8. Factor in resources: Time and costs should be factored in for all required resources, including your data management!
9. Future proof your data: Metadata alone will not future proof your data, you should get DOI’s for your datasets and include relevant README’s and description files.
10. Communicate: If you are working on collaborative research projects then communication is key both in setting up the initial organisational strategies, and throughout the entire project life cycle to ensure that team members are working consistently with respect to data collection, organisation, storage etc.

These tips and the content of this article was collated from our own research, and through the results of running our “Failed it to Nailed it Getting Data Sharing Right” and “Skills4Scientists” series. More information on these series including links to videos can be found here: http://www.ai3sd.org/fi2ni and http://www.ai3sd.org/s4s.

## Data Availability

These tips and the content of this article was collated from our own research, and through the results of running our “Failed it to Nailed it Getting Data Sharing Right” and “Skills4Scientists” series. All referenced videos have already been published on YouTube and deposited in the Southampton ePrints repository with a CC-BY 4.0 License. More information on these series including links to videos can be found here: http://www.ai3sd.org/fi2ni and http://www.ai3sd.org/s4s.

## Abbreviations

RDM: Research data management
FAIR: Findable, accessible, interoperable, reusable
DMP: Data management plan
DOI: Digital object identifier
ELN: Electronic lab notebook
